# Attitudes and influencing factors of nursing assistants towards hospice and palliative care nursing in chinese nursing homes: a cross-sectional study

**DOI:** 10.1186/s12904-023-01175-8

**Published:** 2023-04-25

**Authors:** Zhuojun Ye, Limei Jing, Haoyu Zhang, Yongfa Qin, Hangqi Chen, Jiying Yang, Ruize Zhu, Jingrong Wang, Huiwen Zhang, Yifan Xu, Tianshu Chu

**Affiliations:** School of Public Health, Shanghai University of Traditional Chinese Medicine, Cailun Rd#1200, Pudong New Area, Shanghai, 201203 China

**Keywords:** Hospice and palliative care nursing, Nursing homes, Nursing assistants, Attitudes, Cross-sectional study

## Abstract

**Background:**

Hospice and palliative care nursing (HPCN) in China is mainly available at public primary care institutions, where nursing homes (NHs) are rarely involved. Nursing assistants (NAs) play an essential role in HPCN multidisciplinary teams, but little is known about their attitudes towards HPCN and related factors.

**Methods:**

A cross-sectional study was designed to evaluate NAs’ attitudes towards HPCN with an indigenised scale in Shanghai. A total of 165 formal NAs were recruited from 3 urban and 2 suburban NHs between October 2021 and January 2022. The questionnaire was composed of four parts: demographic characteristics, attitudes (20 items with four sub-concepts), knowledge (nine items), and training needs (nine items). Descriptive statistics, independent samples t-test, one-way ANOVA, Pearson’s correlation, and multiple linear regression were performed to analyse NAs’ attitudes, influencing factors, and their correlations.

**Results:**

A total of 156 questionnaires were valid. The mean score of attitudes was 72.44 ± 9.56 (range:55–99), with a mean item score of 3.6 ± 0.5 (range:1–5). The highest score rate was “perception of the benefits for the life quality promotion” (81.23%), and the lowest score rate was “perception of the threats from the worsening conditions of advanced patients” (59.92%). NAs’ attitudes towards HPCN were positively correlated with their knowledge score (*r* = 0.46, *P* < 0.01) and training needs (*r* = 0.33, *P* < 0.01). Marital status (*β* = 0.185), previous training experience (*β* = 0.201), location of NHs (*β* = 0.193), knowledge (*β* = 0.294), and training needs (*β* = 0.157) for HPCN constituted significant predictors of attitudes (*P* < 0.05), which explained 30.8% of the overall variance.

**Conclusion:**

NAs’ attitudes towards HPCN were moderate, but their knowledge should be improved. Targeted training is highly recommended to improve the participation of positive and enabled NAs and to promote high-quality universal coverage of HPCN in NHs.

**Supplementary Information:**

The online version contains supplementary material available at 10.1186/s12904-023-01175-8.

## Background

Hospice and palliative care nursing (HPCN) should be integrated into nursing services to improve the quality of life for the elderly with the tough challenge of the ageing population. In China, more than 264 million adults were aged 60 years old and older in 2020, which is more than 18.70% of the total population [1]. To meet the growing demand for elderly care, nursing homes (NHs) are becoming increasingly common locations for end-of-life (EOL) care [2]; more than one in five deaths globally occurs in NHs [3]. Over the past decade, the number of NHs in China has increased more than fifteen-fold, with approximately 758 currently [4]. This may be related to an increasing elderly population living alone, expanded life expectancy, and weakening of home-based care [2]. Thus, HPCN has been widely advocated in many countries to improve the quality of life for NH residents [5].

NHs mainly serve older, dependent adults in need of complex care with incurable chronic illness, advanced cancer, cognitive impairment, trauma, and other health conditions [6]. Although death is not necessarily imminent, many inpatients require HPCN upon admission because of mortality trajectories associated with chronic disease and frailty [7]. HPCN can improve quality of life for patients and their families by preventing and relieving suffering, reducing the side effects of treatment, and providing social, psychological, and emotional support. As more than 40 million (18%) of the elderly in China are disabled and semi-disabled [8], the ever-growing demand for NHs has increased the need for HPCN [3]. Researchers have suggested that HPCN in NHs are associated with resident and family satisfaction, rational allocation of resources, and lower hospitalisation rates [9–11].

The significance of HPCN in NHs is widely recognised by managers, nursing staff, residents, and family members [12–15]. However, HPCN in China is mainly available at public primary care institutions and a few secondary and tertiary hospitals; NHs are rarely involved [16]. Concern about HPCN delivery in NHs has aroused global awareness as several barriers to care have been identified. For instance, most NH residents’ deaths are unpredictable, making it difficult to determine the appropriate time to provide HPCN [17]. In addition, declines in communication skills and cognitive function often prevent NH residents from expressing their needs accurately [18]. Knowledge deficit is another main challenge for providing HPCN [19], making early integration of HPCN particularly difficult.

Globally, the shortages of qualified nurses and increased demands associated with the ageing population have led to an increase in unregulated healthcare workers and related services [20]. In NH settings, nursing assistants (NAs) are the primary workforce; they provide most life care, such as helping with feeding, excreting, bathing, and communication, and NAs are the people residents see and interact with the most [21]. In addition, NAs offer spiritual support while communicating with family members and strengthening bonds among residents, family members, and medical staff. Overall, NAs are best placed to identify and support residents’ diverse needs [22] and in the absence of multidisciplinary professionals, play multiple roles in HPCN teams [23].

In China, most NAs are organised by companies without unified management standards or comprehensive training mechanisms [24]. Despite the variation in education, training and qualifications across countries, inadequate training for NAs become a global concern [20], particularly for HPCN. Poor care may aggravate the recurrence and deterioration of diseases, and even cause adverse events, creating a weak point in current elderly care [25]. The importance of HPCN is gradually being recognised, bringing with it demands for competent NAs with favourable attitudes, better knowledge, and the skills necessary to provide high-quality services; these changes can improve residents’ quality of life [26, 27].

However, relatively few studies have assessed the current HPCN attitudes of NAs, which may hinder the extension of HPCN to NHs. Given this gap, we introduced a scientific and indigenised scale to evaluate HPCN attitudes among NAs in NHs, identify associated influencing factors, better understand issues that affect NAs’ attitudes; we hope to facilitate the extension of HPCN and propose countermeasures and suggestions for targeted training to promote the provision of high-quality HPCN in NHs.

## Methods

### Study design and aims

A cross-sectional study was conducted to evaluate NAs’ HPCN attitudes and their associated factors.

### Setting and sample

Using purposive sampling, we surveyed five NHs from five districts in Shanghai, including three urban and two suburban areas and two private and three public NHs. Based on the principles of informed consent, voluntariness, and anonymity, NAs were recruited from the selected NHs. Considering that the sample size should be 5 to 10 times the number of items (20 items in the attitude scale), a total of 165 questionnaires were distributed. The inclusion criteria were: (1) formal NAs, (2) having a minimum of one year of experience in NHs, and (3) fully understanding the content of the questionnaire. The exclusion criteria were NAs who were on leave during the investigation or who were reluctant to participate. After excluding invalid questionnaires, 156 questionnaires were analysed, with an effective response rate of 94.55%.

### Measurements

NAs’ attitudes and related indicators were measured using a scientific scale modified by Shu et al. [28] and Jing et al. [29] that was based on the Frommelt Attitude Toward Care of the Dying Scale (FATCOD) [30] as well as Liu et al.’s attitude questionnaire [31]. In the original study [29], the scale was proven to have good reliability and validity, with a Cronbach’s alpha reliability coefficient of 0.768 and a chi-square value of KMO 0.624. In this study, Cronbach’s alpha was 0.785, and the KMO was 0.776. The questionnaire was composed of four parts: demographic characteristics, attitudes (20 items with four sub-concepts), knowledge (nine items), and training needs (nine items).

The demographic section comprised 13 questions, including age, gender, marital status, educational level, religious beliefs, professional title, HPCN-related experience, and location and type of NHs. The attitude questionnaire consisted of four sub-concepts: perception of the threats from the worsening conditions of advanced patients (four items), perception of the benefits for the life quality promotion (four items), perception of the benefits for better death preparation (four items), and perception of the barriers to providing hospice care (four items). Each item was rated on a 5-point Likert scale ranging from 1 (strongly disagree) to 5 (strongly agree), and the total score ranged from 20 to 100. Negative items (sub-concepts 1 and 4) were reverse-scored. A higher score signified more positive attitudes towards HPCN. Score rate = actual score / total score × 100%. The Knowledge section included nine items. Each item was answered with 1 (true), 2 (false), or 3 (uncertain). Responses were scored as 1 (correct) or 0 (incorrect), and the total score ranged 0–9. A higher score indicated a better understanding of knowledge. The section on training needs contained nine items using a five-point Likert scale. Responses ranged from “completely unnecessary” to “completely necessary” on a scale of 1 to 5, respectively. A higher score indicated a more urgent need for HPCN training.

### Data collection procedure

Data collection occurred between October 2021 and January 2022. Electronic questionnaires were issued to NAs who could read and complete the questionnaire independently, and face-to-face questionnaires were used for those who could not complete the questionnaire independently. Participants were given written or oral explanations of the purpose and procedures of the study. After the data collection, the quality controllers performed a logic check.

### Data analysis

Means and SD were used to describe continuous variables, and frequencies (n) and percentages (%) were calculated for categorical variables. The data analysis consisted of two parts. Potential factors influencing attitudes were first identified using independent-sample t-tests and one-way ANOVA. Pearson’s correlations were performed to examine the relationships between attitudes, knowledge, and training needs. Next, the attitude score was treated as a continuous variable in a multiple linear regression analysis using all factors significantly associated with the attitudes. The significance level was set at 0.05 (2-tailed). All data were analysed using Statistical Package for the Social Sciences (SPSS, version 23.0).

## Results

### General information and characteristics of participants

Most of the 156 NAs were female (84%). The mean age was 50.8 years (range 30–67 years), with 74.4% aged over 50 years. The majority had graduated from junior high school and below (78.8%). For marital status, 93.6% of participants were either married or unmarried. More than half had professional titles (64.7%), and 80.8% reported no religious beliefs. Approximately 85% of respondents had experienced caring for dying residents or family members and witnessed the death process of EOL residents, and about 70% provided HPCN for residents. In addition, nearly half (49.4%) were exposed to knowledge related to HPCN, and 37.8% reported previous training experience with HPCN. Among all participants, 120 NAs (76.9%) were from urban NHs and 52.6% were from private NHs **(**Table 1**)**.

**Table 1:** Demographic characteristics and general information of participants (N = 156)

Variable	Category	N (%)	Attitudes towards HPCN		
			Mean ± SD	*t/F*	*P*
Age (years)	< 40	4 (2.6)	78.50 ± 3.40	0.930	0.397
	40–49	36 (23.1)	71.64 ± 1.55		
	≥ 50	116 (74.4)	72.47 ± 0.90		
Gender	Male	25 (16.0)	74.12 ± 1.84	0.961	0.338
	Female	131 (84.0)	72.11 ± 0.84		
Marital status	Unmarried or married	146 (93.6)	71.88 ± 0.73	-2.855	0.005
	Divorced or widowed	10 (6.4)	80.60 ± 4.88		
Educational level	Junior high school and below	123 (78.8)	71.96 ± 0.88	1.864	0.159
	High school	27 (17.3)	73.04 ± 1.41		
	University and above	5 (3.2)	79.50 ± 5.50		
Religious belief	Yes	30 (19.2)	73.67 ± 1.88	-0.784	0.435
	No	126 (80.8)	72.14 ± 0.84		
Professional title	Yes	101 (64.7)	73.48 ± 0.95	-1.854	0.066
	No	55 (35.5)	70.53 ± 1.27		
Nursing experience for dying residents/ family members	Yes	133 (85.3)	73.05 ± 0.84	-1.930	0.055
	No	23 (14.7)	68.91 ± 1.73		
Witness of death process of EOL residents	Yes	134 (85.9)	72.81 ± 0.84	-1.219	0.225
	No	22 (14.1)	70.14 ± 1.77		
Provision of HPCN	Yes	111 (71.2)	72.98 ± 0.94	-1.121	0.264
	No	45 (28.8)	71.09 ± 1.31		
Exposure to related knowledge of HPCN	Yes	77 (49.4)	74.47 ± 1.04	-2.671	0.008
	No	79 (50.6)	70.46 ± 1.08		
Training experience of HPCN	Yes	59 (37.8)	75.88 ± 1.20	-3.647	< 0.001
	No	97 (62.2)	70.34 ± 0.93		
location of NHs	Urban	120 (76.9)	71.27 ± 0.82	-2.852	0.005
	Suburban	36 (23.1)	76.33 ± 1.76		
Type of NHs	Public	74 (47.4)	73.36 ± 1.11	1.154	0.250
	Private	82 (52.6)	71.60 ± 1.06		

HPCN, hospice and palliative care nursing; NH, nursing home.

### Attitudes towards HPCN

The mean attitude score of respondents was 72.44 ± 9.56 (range: 55–99) out of 100, with the mean item score of 3.6 ± 0.5 (range: 1–5). Sub-concept 2 (perception of the benefits for the life quality promotion) had the highest score (81.23%), and its mean item score was 4.1 ± 0.7; meanwhile, whereas sub-concept 1 (perception of the threats from the worsening conditions of advanced patients) had the lowest score (59.92%), and its mean item score was 3.0 ± 0.9. When examining each item separately, the most positive item was item 14: ‘HPCN can help medical staff to take care of patients better’ (4.26 ± 0.85, 85.12%), and the most negative item was item 2: ‘advanced patients with the worsening conditions are hopeless for the cure’ (2.80 ± 1.16, 55.90%) **(**Table 2**)**.

**Table 2:** Item scores of attitudes regarding HPCN (N = 156)

Dimensions and Items	Score Point $\stackrel{-}{(x}$*±s*)	Scoring Rate (%)
**Perception of the threats from the worsening conditions of advanced patients is**:	15.0 ± 4.30	59.92
1. Uncomfortable to take care of advanced cancer patients.	3.34 ± 1.41	66.80
2. Hopeless for the cure.	2.80 ± 1.16	55.90
3. Unable to easily face dying process and distress.	2.94 ± 1.32	58.84
4. Make me often think about death.	3.08 ± 1.33	61.54
5. Make me feel weakness.	2.83 ± 1.37	56.54
**Perception of the benefits for the life quality promotion is**:	20.3 ± 3.50	81.23
6. Able to promote life quality.	3.96 ± 0.95	79.24
7. Able to die peacefully and have a good death.	4.19 ± 0.90	83.84
8. Able to relieve pain and other symptoms.	4.00 ± 0.89	80.00
9. Emotional support.	4.06 ± 0.89	81.16
10. Able to have family support.	4.10 ± 0.79	81.92
**Perception of the benefits for better death preparation is**:	19.4 ± 3.20	77.64
11. Respect for patient’s religion and burial rites.	3.93 ± 0.90	78.58
12. Help to die at home.	3.59 ± 1.00	71.80
13. Better communication with advanced patients.	4.16 ± 0.86	83.20
14. Help medical staff to take care of patients better.	4.26 ± 0.85	85.12
15. Avoid the idea of euthanasia.	3.47 ± 1.09	69.48
**Perception of the barriers to providing hospice care is**:	17.7 ± 3.50	70.95
16. Feel like euthanasia.	3.42 ± 1.12	68.34
17. No active treatment for physical symptoms but just waiting for death.	3.73 ± 1.09	74.62
18. Mean giving up patients.	3.92 ± 1.10	78.34
19. Make patients feel hopeless.	3.86 ± 1.03	77.18
20. Advanced patients have many difficult symptoms.	2.81 ± 1.21	56.28
**Total Points**	72.44 ± 9.56	72.44

### Factors influencing attitudes regarding HPCN

The average response accuracy of HPCN knowledge was 61.1%, with a total score of 5.5 ± 2.3 (range: 0–9); but the training needs section was scored as 34.43 ± 6.95 (range: 9 ~ 45), with a proportion of 95.5%. A moderate positive correlation showed that attitudes towards HPCN were more positive as knowledge scores (*r* = 0.46, *P* < 0.01) and training needs increased (*r* = 0.33, *P* < 0.01). The univariate analysis suggested that better attitudes were associated with a marital status of divorced or widowed (*P* < 0.01), exposure to related knowledge of HPCN (*P* < 0.01), training experience in HPCN (*P* < 0.001), and suburban location (*P* < 0.01) **(**Table 1**)**. To identify the predictors of NHs’ attitudes towards HPCN, variables with statistical significance were entered into the multiple linear regression model. The significant predictors of attitudes were marital status (*β* = 0.185), training experience in HPCN (*β* = 0.201), location of NHs (*β* = 0.193), knowledge of HPCN (*β* = 0.294), and training needs for HPCN (*β* = 0.157), which explained 30.8% of the variance (adjusted R^2^ = 0.308, *P* < 0.05). Being divorced or widowed, training experience in HPCN, better HPCN knowledge level, higher HPCN training needs, and suburban location were all significantly and positively associated with attitude scores **(**Table 3**)**.

**Table 3:** Multiple linear regression analysis of factors influencing the attitudes of NAs towards HPCN (N = 156)

Variables	B	*S.E.*	*β*	*t*	*P*	Tolerance	VIF	*R* ^*2*^	Adj *R*^*2*^
Constant	55.257	3.289	-	16.801	< 0.001			0.330	0.308
Marital status									
Unmarried or married	Reference								
Divorced or widowed	7.180	2.622	0.185	2.739	0.007	0.984	1.016		
Training experience of HPCN									
NO	Reference								
YES	3.959	1.350	0.201	2.932	0.004	0.946	1.057		
location of NHs									
Urban	Reference								
Suburban	4.364	1.553	0.193	2.811	0.006	0.948	1.055		
Knowledge of HPCN	1.229	0.339	0.294	3.630	< 0.001	0.679	1.472		
Training needs for HPCN	0.217	0.109	0.157	1.979	< 0.05	0.705	1.418		

HPCN, hospice and palliative care nursing; NH, nursing home.

## Discussion

To our knowledge, this is the first study to investigate the attitudes and factors related to HPCN among NAs in NH settings in China. Although an increasing number of older adults need high-quality HPCN in NHs, and NAs are the main providers, we found that limitations still exist in their attitudes and knowledge regarding HPCN. The findings will not only clarify NAs’ attitudes towards HPCN but also suggest targeted training and enabling facilitators for the extension of HPCN to NHs, in which HPCN should be provided but rarely involved. Policymakers can formulate policies based on the results of this study to ensure high-quality HPCN for all EOL elderly patients.

Our study revealed that NAs in NHs had moderate attitudes towards HPCN. The overall scoring rate was 72.44%, which is slightly lower than that reported in a previous study in Shanghai (volunteers of HPCN, 74.54%) [29] and in Taiwan (long-term care staff of advanced dementia, 73.7%) [32]; however, the rate was higher than a study conducted in Canada (long-term care workers of EOL palliative care, 70.6%) [33]. These differences are likely attributable to international cultural differences regarding life and death, related training, lack of related studies on NAs, and differences in scales.

Generally, NAs fully recognised the benefits of HPCN in NH settings. They agreed that HPCN could positively affect EOL residents, reduce pain, and enhance dignity. Nevertheless, most NAs felt negatively when faced with the worsening conditions of advanced residents and were unable to face the dying process and distress, which is consistent with previous findings [34]. This may be related to death taboos in China. Participants in this study provide personal care for terminal residents in NHs, where deaths occur regularly and are prone to negative emotions, which makes it difficult for NAs to provide high-quality care. Furthermore, these negative views may affect the motivation of NAs to continue their nursing careers and have profound consequences for the provision and extension of HPCN.

In terms of NAs’ knowledge of HPCN, the average response accuracy of HPCN knowledge was 61.1%, which is significantly lower than that of a Chinese study involving volunteers (82.1%) [29], a Taiwanese study involving long-term care staff (62.3%) [32], and an Australian study involving dementia care staff (62.5%) [35]. The results highlight that NAs’ knowledge was disproportionate to their important role in the team, impeding their ability to provide high-quality HPCN in NHs, and negatively impacting residents’ EOL quality. This may be related to the older age and lower educational level of the participants involved in this study, in keeping with the general situation of Chinese NAs. The difficulty in accepting and independently updating professional knowledge has become a shortcoming for NAs. This may also be due to the lack of HPCN training in China, where related knowledge has not yet been included in professional qualification training for NAs, consistent with the result that less than half of the involved NAs have been exposed to relevant knowledge.

There was a significant positive correlation between knowledge and attitudes, and knowledge was shown to be the strongest factor associated with attitudes, which has also been reported in other studies [32, 36]. The results indicate that better knowledge can help improve NHs’ attitudes towards HPCN. This may be explained by the construction of self-confidence through knowledge acquisition. Previous research demonstrated that NAs often lack confidence and feel uncomfortable in providing HPCN when they lack sufficient knowledge, which negatively influences their work performance, levels of work stress, ability to provide health guidance to residents, sensitivity to residents’ and families’ care needs, and their HPCN attitudes [36, 37].

HPCN attitudes were also associated with NAs’ marital statuses. NAs who were divorced or widowed reported better attitudes towards HPCN, differing from other studies that focused on practitioners and nurses and conducted in tertiary hospitals in China, which indicated that married medical staff had more positive attitudes [38, 39]. Being divorced or widowed is a significant trigger of empathy. Such experiences allow NAs to share the pain of the death of a loved one, creating a deep understanding of the HPCN, and is assumed to demystify the dying process and thereby reduce negative emotions. This matches the evidence that those who have already been exposed to death have more positive attitudes towards HPCN [40].

NAs from suburban NHs showed more positive attitudes towards HPCN than those from urban areas. This result has rarely been reported in previous studies. This may be related to the strong sense of community and mutual assistance in suburban communities, which helps develop harmonious communication and intimate emotional bonds through daily contact and caregiving. Our study revealed that there may be an opportunity to improve attitudes through better communication, highlighting the importance of excellent bedside communication skills with EOL residents.

Having HPCN training had a positive impact on attitudes, indicating that a lack of professional training in HPCN will inevitably affect NAs’ attitudes. Furthermore, we found that over 95.5% of NAs reported urgent training needs for HPCN, similar to previous studies in NH settings [3, 32]. Considering that NAs lack standardised training and learn exclusively by observing and imitating more experienced colleagues [41], this can be considered a reflection of NAs feeling intense support for EOL care issues, which was confirmed by international studies [42, 43]. NAs with higher training needs showed better attitudes regarding HPCN than those with lower training needs; those with higher training needs were more likely to believe in the benefits of HPCN for EOL residents and were thus more eager to acquire relevant knowledge that would allow them to provide quality HPCN.

Favourable attitude is considered the most significant enabler in providing high-quality HPCN services [38], which can be achieved through targeted training and practical experience [44]. In the context of the extensive pilot reforms of elderly care and HPCN in China, the construction of a theoretical and practical training system for NAs is still under exploration. Furthermore, there is still a lack of corresponding regulations regarding how to integrate the concept and practice of HPCN into the professional skill standards of NAs. Therefore, it is urgent to promote professional and targeted training tailored to their characteristics and acceptability based on their knowledge deficiency, moderate attitudes, and training needs, which can rationally and perceptually improve NAs’ HPCN attitudes. Additionally, the HPCN concept and related knowledge should be incorporated into the qualification training of NAs to help equip them with essential knowledge, full self-confidence, abundant first-hand experience, and excellent bedside communication skills for EOL residents.

## Limitations

There were several limitations in our study. First, the data were drawn from a small purposive sample of participants, which may potentially introduce selection bias and limit the generalisability of our findings. Second, this is a cross-sectional study, showing only correlations but no causal relationship between attitudes towards HPCN and the factors mentioned above, so further longitudinal research is needed to evaluate these associations. Finally, based on the self-reported questionnaire, information bias may have occurred. Additionally, due to the limited length of the questionnaire, only a few influencing factors were conducted in this study. It is worthwhile to include factors like years of work experience, income, and physical conditions so as to find more effective ways to tailor targeted training programs.

## Conclusions

In China, the government is trying to extend HPCN to NHs and strengthen multidisciplinary teams. This study found that NAs showed moderate attitudes towards HPCN, which improved as their knowledge score increased and training needs progressed. In addition, better HPCN attitudes were significantly associated with being divorced or widowed, training experience of HPCN, and suburban location. Integrated and continuous HPCN in NHs can be achieved only with the participation of positive and enabled NAs. Thus, there is an urgent need to scale up targeted training for NAs with different characteristics and incorporate concepts and knowledge of HPCN into professional qualification training to help improve their attitudes towards HPCN and equip them with essential knowledge and skills. The results of this study will help promote high-quality universal coverage of HPCN in NHs and enlighten other regions and countries with the same level of domestic and international development.

## Electronic supplementary material

Below is the link to the electronic supplementary material.

Appendix: Nursing Assistants’ Attitudes, Knowledge and Training Needs of Hospice and Palliative Care Nursing Scale

## Data Availability

All datasets during and/or analysed during this study are available from the corresponding author upon reasonable request.

## List of abbreviations

HPCN: Hospice and palliative care nursing
NHs: Nursing homes
NAs: Nursing assistants
EOL: End of life.
