# Supplementary material for: Attitudes and influencing factors of nursing assistants towards hospice and palliative care nursing in chinese nursing homes: a cross-sectional study
Source: BMC Palliat Care. 2023 Apr 25;22:49. doi: 10.1186/s12904-023-01175-8 (PMC10127064; doi:10.1186/s12904-023-01175-8)
Supplement: Supplementary file 1 — Appendix: Nursing Assistants’ Attitudes, Knowledge and Training Needs of Hospice and Palliative Care Nursing Scale [file 12904_2023_1175_MOESM1_ESM.docx]

**Appendix:**

**Nursing Assistants' Attitudes, Knowledge and Training Needs of Hospice and Palliative Care Nursing Scale**

In the context of the extensive pilot reforms of elderly care and hospice and palliative care nursing (HPCN) in China, nursing homes are vital force in the provision of related services. This scale is to survey your basic status of Attitudes, Knowledge and Training Needs of HPCN to provide the scientific evidence for guiding the targeted training and related practices of HPCN for nursing assistants in nursing home settings. The questionnaire is voluntary and anonymous. It may cost you 5-10 min. Thanks for your support and cooperation.

**Demographic Sheet**

1. Institution name: ____________
2. Age: ____________ (years old)
3. Gender: ○Male ○Female
4. Marital status: ○Unmarried or married ○Divorced or widowed
5. Educational level: ○Junior high school and below ○High school ○University and above
6. Have you had a religious belief? ○Yes ○No
7. Have you had a professional title? ○Yes ○No
8. Have you had nursing experience for dying residents/ family member? ○Yes ○No
9. Have you witnessed the death process of end-of-life residents? ○Yes ○No
10. Have you provided any HPCN? ○Yes ○No
11. Have you exposed to related knowledge of HPCN? ○Yes ○No
12. Have you had any training experience in HPCN before? ○Yes ○No

| **Attitudes Sheet** | | | | | | | | | | | |
| --- | --- | --- | --- | --- | --- | --- | --- | --- | --- | --- | --- |
| Which answer corresponds to your own personal feelings about the attitude presented, please fill in the corresponding number in the blank. The meaning of number is:  1=totally disagree, 2=partly disagree, 3= neutral/nonsense, 4=partly agree, 5= totally agree. | | | | | | | | | | | |
| **Dimensions and Items** | | | **1** | | **2** | | | **3** | | **4** | **5** |
| **Perception of the threats from the worsening conditions of advanced patients is:** | | | | | | | | | | | |
| 1. Uncomfortable to take care of advanced cancer patients. | | |  | |  | | |  | |  |  |
| 2. Hopeless for the cure. | | |  | |  | | |  | |  |  |
| 3. Unable to easily face dying process and distress. | | |  | |  | | |  | |  |  |
| 4. Make me often think about death. | | |  | |  | | |  | |  |  |
| 5. Make me feel weakness. | | |  | |  | | |  | |  |  |
| **Perception of the benefits for the life quality promotion is:** | | | | | | | | | | | |
| 6. Able to promote life quality. | | |  | |  | | |  | |  |  |
| 7. Able to die peacefully and have a good death. | | |  | |  | | |  | |  |  |
| 8. Able to relieve pain and other symptoms. | | |  | |  | | |  | |  |  |
| 9. Emotional support. | | |  | |  | | |  | |  |  |
| 10. Able to have family support. | | |  | |  | | |  | |  |  |
| **Perception of the benefits for better death preparation is:** | | | | | | | | | | | |
| 11. Respect for patient's religion and burial rites. | | |  | |  | | |  | |  |  |
| 12. Help to die at home. | | |  | |  | | |  | |  |  |
| 13. Better communication with advanced patients. | | |  | |  | | |  | |  |  |
| 14. Help medical staff to take care of patients better. | | |  | |  | | |  | |  |  |
| 15. Avoid the idea of euthanasia. | | |  | |  | | |  | |  |  |
| **Perception of the barriers to providing hospice care is:** | | | | | | | | | | | |
| 16. Feel like euthanasia. | | |  | |  | | |  | |  |  |
| 17. No active treatment for physical symptoms but just waiting for death. | | |  | |  | | |  | |  |  |
| 18. Mean giving up patients. | | |  | |  | | |  | |  |  |
| 19. Make patients feel hopeless. | | |  | |  | | |  | |  |  |
| 20. Advanced patients have many difficult symptoms. | | |  | |  | | |  | |  |  |
| **Knowledge Sheet** | | | | | | | | | | | |
| Please indicate the right answer and fill in the corresponding number. | | | | | | | | | | | |
| **Items** | **Option** | | | | | | | | | | |
|  | **Yes** | **No** | | | | | **Don't know** | | | | |
| 1. HPCN is respecting natural death, but not shortening the life of patients. |  |  | | | | |  | | | | |
| 2. Psychological, social, and spiritual problems are paramount to the HPCN team who give appropriate consultation and management. |  |  | | | | |  | | | | |
| 3. Community resource allocation for patients and families is also part of HPCN. |  |  | | | | |  | | | | |
| 4. HPCN is interdisciplinary work. |  |  | | | | |  | | | | |
| 5. Home care for terminally ill patients is not part of HPCN. |  |  | | | | |  | | | | |
| 6. Pain and other physical symptoms in terminally ill patients can be managed with high-quality HPCN. |  |  | | | | |  | | | | |
| 7. The HPCN team provides bereavement support for the family after the patient's death. |  |  | | | | |  | | | | |
| 8. Helping patients face and prepare for death is part of HPCN. |  |  | | | | |  | | | | |
| 9. HPCN respects the freedom and individual needs of patients. |  |  | | | | |  | | | | |
| **Training Needs Sheet** | | | | | | | | | | | |
| Which answer matches your personal needs for the training program presented, please fill in the corresponding number in the blank. The meaning of the numbers is:  1=Not at all, 2= Not very necessary, 3= General, 4= Somewhat necessary, 5= Very necessary | | | | | | | | | | | |
| **Items** | | | | **1** | | **2** | | | **3** | **4** | **5** |
| 1. Death education and life care. | | | |  | |  | | |  |  |  |
| 2. Utilization of medical resources and community health services. | | | |  | |  | | |  |  |  |
| 3. Main content of HPCN. | | | |  | |  | | |  |  |  |
| 4. Domestic and foreign experience and practices of HPCN. | | | |  | |  | | |  |  |  |
| 5. Contents and methods of spiritual care. | | | |  | |  | | |  |  |  |
| 6. Living will and HPCN Act. | | | |  | |  | | |  |  |  |
| 7. Social support and utilization of community resources. | | | |  | |  | | |  |  |  |
| 8. The management method of the patient's physical symptoms. | | | |  | |  | | |  |  |  |
| 9. Methods and skills of communicating with terminal patients and their families. | | | |  | |  | | |  |  |  |
